# Molecular Mechanisms of Oligodendrocyte Regeneration in White Matter-Related Diseases

**DOI:** 10.3390/ijms19061743

**Published:** 2018-06-12

**Authors:** Ryo Ohtomo, Atsushi Iwata, Ken Arai

**Affiliations:** 1 Neuroprotection Research Laboratory, Departments of Radiology and Neurology, Massachusetts General Hospital and Harvard Medical School, Charlestown, MA 02129, USA; ROHTOMO@mgh.harvard.edu; 2 Department of Neurology, The University of Tokyo Graduate School of Medicine, Tokyo 113-8654, Japan; Iwata-tky@umin.ac.jp

**Keywords:** neurovascular unit, vascular cognitive impairment syndrome, Alzheimer’s disease, multiple sclerosis, white matter degeneration, remyelination, oligodendrocyte precursor cell

## Abstract

Even in adult brains, restorative mechanisms are still retained to maintain the microenvironment. Under the pathological conditions of central nervous system (CNS) diseases, several immature cells in the brain would be activated as a compensative response. As the concept of the neurovascular unit emphasizes, cell-cell interactions play important roles in this restorative process. White matter damage and oligodendrocyte loss are representative characteristics for many neurodegenerative diseases. In response to oligodendrocyte damage, residual oligodendrocyte precursor cells (OPCs) initiate their proliferation and differentiation for the purpose of remyelination. Although mechanisms of oligodendrogenesis and remyelination in CNS diseases are still mostly unknown and understudied, accumulated evidence now suggests that support from neighboring cells is necessary for OPC proliferation and differentiation. In this review, we first overview basic mechanisms of interaction between oligodendrocyte lineage cells and neighboring cells, and then introduce how oligodendrogenesis occurs under the conditions of neurodegenerative diseases, focusing on vascular cognitive impairment syndrome, Alzheimer’s disease, and multiple sclerosis.

## 1. Introduction

**Figure 1:**
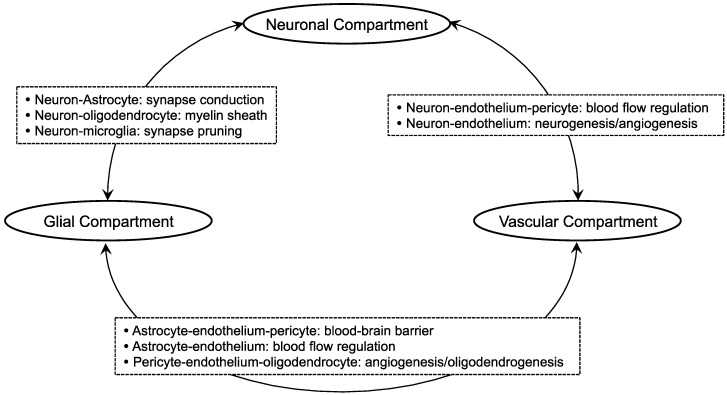
Schematic of the neurovascular unit (NVU): One of the key messages from the concept of the neurovascular unit may be that cell-cell interaction is critical for maintaining brain function. This schematic introduces some examples of how each component works together in the neurovascular unit. The neuronal compartment may include neuronal precursor cells along with neurons. The glial compartment includes astrocytes, oligodendrocytes, and microglia. The vascular compartment may include both endothelium and pericytes. Please note that there are several important mechanisms of cell-cell interaction even within each compartment. For example, in the glial compartment, microglia regulate the phenotypic change in astrocytes [7]. Also in the same compartment, oligodendrocytes interact with astrocytes and microglia.

It has been nearly 20 years since the neurovascular unit (NVU), a conceptual structural unit composed of neurons, glial cells (astrocytes, microglia, oligodendrocytes), vascular endothelial/smooth muscle cells, pericytes, and extracellular matrix, was proposed as a new paradigm for the investigation of stroke [1,2]. The emergence of this concept was based on the fact that brain function along with dysfunction arise from integrated interactions between networks of cellular components as listed above (Figure 1). Since NVU is responsible for the regulation of blood flow and blood-brain barrier (BBB) through the vascular system, the concept of NVU has been utilized to elucidate pathological mechanisms of other neurological diseases (partly/totally) caused by deficient cerebral blood flow (CBF) or the breakdown of BBB [3,4,5,6]. NVU has broadened our knowledge to manage complex diseases by unveiling a variety of novel findings about cell-cell and cell-extracellular matrix interactions that occur during the disease course. As primary etiologies for central nervous system (CNS) diseases are most likely to be found in the gray matter, past studies mainly focused on the protection and recovery of neurons that lie within the gray matter. However, since alterations of the white matter are observed in those diseases as well, referring to white matter components as a therapeutic target should also be of great importance.

Oligodendrocyte, categorized as a sub-type of glial cells, is one of the major cell types in the cerebral white matter. Oligodendrocytes contribute to the white matter function by forming myelin sheaths. As the concept of NVU emphasizes, oligodendrocytes may not stand alone and should receive support from neighboring cells to play their roles in the white matter. Therefore, clarifying non-cell autonomous mechanisms of oligodendrocyte regeneration should be essential to understand the pathophysiology of the white matter in the recovery phase of neurodegenerative diseases. In this review, we will first briefly overview oligodendrocyte-related cell-cell interaction in the white matter. Then, we will discuss how those cell-cell interactions are involved in oligodendrocyte damage/regeneration under the diseased conditions of CNS diseases, such as vascular cognitive impairment syndrome (VCI, formerly called vascular dementia), Alzheimer’s disease (AD), and multiple sclerosis (MS, a representative white matter disease).

## 2. Oligodendrocyte-Related Cell-Cell Interaction in White Matter

The main components of the white matter are neuronal axons, oligodendrocyte lineage cells (e.g., oligodendrocytes and oligodendrocyte precursor cells (OPCs)), endothelial cells, astrocytes, and microglia. These cells may support each other to maintain white matter homeostasis. However, compared to the mechanisms of cell-cell interaction in the gray matter, the NVU function/role in the white matter is still relatively unknown. Nonetheless, recent studies have revealed many key mechanisms for cell-cell interaction in the white matter, especially under the pathophysiological conditions of neurodegenerative diseases. One of the main differences in the NVU between the gray matter and the white matter may be the involvement of oligodendrocyte lineage cells. Therefore, in order to consider effective therapeutic approaches for white matter-related diseases, we may need to understand how oligodendrocyte lineage cells interact with other surrounding cells. 

A well-documented oligodendrocyte-related cell-cell interaction is in myelinated axons. Myelin sheaths formed by oligodendrocytes are critical in increasing impulse speed to achieve fast and effective neuronal signal transduction. Furthermore, through a myelin-independent manner, oligodendrocytes can maintain the functional integrity and survival of axons. For example, oligodendrocytes secrete several trophic factors, such as insulin-like growth factor-1 (IGF-1) and glial-cell derived neurotrophic factor (GDNF), to support axonal function [8]. Conversely, axonal activities may in turn help oligodendrocyte lineage cells. Axon-secreted molecules or axonal surface ligands have been reported to regulate the differentiation and maturation processes of oligodendrocytes. For example, Jagged ligands expressed in axons send signal to OPCs through the Notch pathway to inhibit their differentiation [9]. Axon-myelin interaction is disturbed under diseased conditions. Just like the gray matter, the activation of several deleterious factors and pathways takes place during the course of degeneration in the white matter. For example, under the acute phase of white matter injury, oligodendrocyte function would be affected/changed by direct attack from deleterious substances such as matrix metalloproteinases (MMPs), while some MMPs would be beneficial during the chronic phase. Also, in a recent study, vesicular glutamate release from axons was shown to cause myelin damage through GluN2C/D-containing *N*-methyl-d-aspartate (NMDA) glutamate receptors [10]. Even if oligodendrocyte (and its precursor) cells could avoid immediate death from those factors, the metabolic dysfunction of oligodendrocyte lineage cells triggered by the assault would cause abnormal myelin replenishment and the synthesis of myelin-related proteins, resulting in an impairment of myelin-axon coupling. Then, eventually, the disturbance of oligodendrocyte-neuron interaction would lead to white matter dysfunction.

Besides neuron-glia interaction, oligodendrocytes also work closely with other sub-types of glial cells. Astrocytes are well known to physically interact with neighboring cells to maintain a strictly-regulated microenvironment in the brain. For example, in the perivascular region, astrocytes tune vascular tone and CBF through their fine processes that form a close liaison with blood vessels [11]. Astrocytes also support vascular endothelial cells to form the BBB to protect brain cells against several deleterious substances. In addition, through gap junctions, astrocytes directly interact with oligodendrocytes to support their function [12,13]. Furthermore, an indirect interaction may also be important for the support from astrocytes to oligodendrocytes. Soluble factors secreted from astrocytes are reported to protect oligodendrocyte lineage cells from external stress such as hypoxia [14]. In an animal model of acute spinal cord injury, transplantation of cultured astrocytes facilitated the myelin restoration in the demyelinated lesion via accelerating the proliferation of endogenous OPCs [15]. Microglia, another sub-type of glial cells, also have an important interaction with oligodendrocyte lineage cells. In a cell-culture system, microglial presence was demonstrated to increase the synthesis of myelin-specific proteins in cultured OPCs [16]. Other studies also confirmed that conditioned medium from non-activated microglia enhanced the survival/maturation of OPCs in vitro [17,18]. These studies used non-activated microglia, but the microglial phenotype may be closely related to regulate OPC function. In a mouse model of traumatic injury, histone deacetylase (HDAC) inhibition prevented white matter damage, possibly through phenotypic switch to the M2 microglia that preserve neighboring oligodendrocyte lineage cells both in vivo and in vitro [19]. Taken together, the interaction with other sub-types of glial cells would be necessary for oligodendrocyte lineage cells to keep their roles both under pathological and physiological conditions.

The cerebrovascular system (e.g., cerebral endothelial cells and pericytes) also provides an important mechanism for oligodendrocyte lineage cells to play roles in white matter. Although a physical contact of mature oligodendrocytes with cerebral endothelial cells has not been confirmed so far, OPCs are predicted to directly contact with cerebral endothelial cells (and pericytes) in the perivascular region [20,21]. OPCs are known to be active during the developmental phase to generate mature oligodendrocytes that would eventually form myelin sheaths. Although the formation of myelinated tracts occurs at the early stage of life, their homeostatic density is maintained throughout life. For myelin homeostasis in an adult brain, neural stem progenitor cells (NSPCs) may be required to transform into OPCs in addition to residual OPCs [22,23,24,25]. In response to white matter injury in adult brains, NSPCs inside the subventricular zone (SVZ) along with the residual OPCs outside the subventricular zone would proliferate and differentiate at a faster rate to promote the endogenous repairing process [26,27,28,29,30]. When OPCs in adult brains are activated in demyelinated lesions, they revert their phenotype to active OPCs that could be found in neonatal brains. They were found to produce cytokine interleukin-1β (IL-1β) and C-C motif chemokine 2 (CCL2) to enhance the mobilization and repopulation of the OPCs for remyelination [31]. NSPC-derived Olig2-expressing cells in the SVZ differentiate into highly migratory OPCs that reside close to the blood vessels. These vessels serve as a scaffold for the migration to the area of injury by releasing chemoattractants such as brain-derived neurotrophic factor (BDNF) and fibroblast growth factor-basic (bFGF) [27,28,32,33]. Past studies using in vitro cell-culture systems carefully examined the involvement of cerebral vascular endothelial cells in regulating the function of oligodendrocyte lineage cells. For example, co-culture of endothelium with NSPCs has shown that the interaction between endothelium and neural precursor cells promotes the differentiation of NSPCs into oligodendrocytes (as well as astrocytes and neurons) under the influence of CCL2/monocyte chemotactic protein-1 (MCP-1) [34]. Another study showed that conditioned media from endothelial cells promoted the transformation of NSPCs into oligodendrocyte lineage cells [35]. In addition, endothelial-derived growth factors are confirmed to promote the proliferation and migration of OPCs in vitro [36,37]. However, the expression pattern of endothelial growth factors may change by oxidative stress [36,38], and stressed endothelial cells could not support the OPC proliferation in vitro [36]. Therefore, the disturbance of OPC-endothelium trophic coupling under pathological conditions may be a part of reasons for myelin degradation or white matter dysfunction. In addition, as stated above, OPCs may also physically come into contact with pericytes in the perivascular region of white matter. Although the mechanism of OPC-pericyte interaction is still mostly unknown, a recent comprehensive study, which conducted magnetic resonance imaging (MRI), viral-based tract-tracing, and behavior and tissue analysis with pericyte-deficient mice, demonstrated that pericyte degeneration disrupted the white matter microcirculation [39]. In addition, the disruption of the microcirculation resulted in an accumulation of toxic blood-derived fibrin (ogen) deposits and CBF reductions, which triggered a loss of myelin, axons, and oligodendrocytes [39]. Another study also confirmed the supportive roles of pericytes in OPCs by showing that conditioned media from pericytes supported the survival of OPCs in vitro [21]. In addition, a recent study indicated an involvement of an anchoring protein A-kinase anchor protein 12 (AKAP12) in the supportive roles of pericytes for OPC function. AKAP12 regulated growth factor production in pericytes and conditioned media from AKAP12-deficient pericytes no longer supported the differentiation of OPCs in vitro [40]. As several types of brain cells interact with each other in the perivascular space [41], future studies are warranted to examine the oligodendrocyte-related cell-cell interaction in the region to obtain a deeper understanding of the pathological mechanisms of neurodegenerative diseases.

## 3. Molecular Mechanisms of Oligodendrocyte Repair/Damage in Neurodegenerative Diseases

Under the pathological conditions of neurodegenerative diseases, multiple deleterious cascades are simultaneously over-activated to exacerbate oligodendrocyte damage. Since it would be difficult to block all of these pathways at once, we may also need to pay attention to the mechanisms of oligodendrocyte regeneration to achieve effective therapy for white matter-related diseases. In this section, by focusing on VCI, AD, and MS, which would be more likely to present vast cerebral white matter lesions compared to other neurodegenerative diseases (except special genetic disorders), we will discuss what kind of mechanisms are involved in oligodendrogenesis under these diseased conditions.

### 3.1. Vascular Cognitive Impairment Syndrome (VCI)

VCI is caused by the impairment of blood supply to the brain. Even though VCI is usually not recognized as a demyelinating disease, white matter dysfunction does occur in most cases of VCI. For example, in the case of subcortical ischemic vascular dementia, which is the most common subtype of VCI [42,43], a pathological background of stepwise-worsening neurological deficits and a loss of executive function represent immense periventricular white matter degeneration due to chronic cerebral hypoperfusion caused by fibrohyalinosis of the medullary artery [44,45,46]. Analyses of postmortem brain from patients with VCI revealed that there was a significant increase of various progenitor cells [47]. In the SVZ and peri-infarct regions, cells that were positive for doublecortin (DCX), nestin, and polysialylated neural cell adhesion molecules (PSA-NCAMs) were accumulated in stroke patients. In addition, in the white matter lesions of VCI patients, OPCs were also increased while mature oligodendrocytes were decreased [47]. These studies may suggest that there is an endogenous mechanism to promote oligodendrogenesis in the brain of VCI patients. However, in most cases, the recovery of lost functions cannot be achieved. This may partially be due to a lack of growth factors that could support the proper differentiation of progenitor cells under the conditions of chronic cerebral hypoperfusion. Also, since the prevalence of VCI increases with age, aging itself may cause negative effects towards white matter regeneration, presumably by poor blood perfusion to the lesion and/or by the absence of systemic factors essential for compensative responses [48].

Thus far, detailed biological mechanisms that suppress oligodendrocyte regeneration in patients with VCI still remain elusive. However, recent basic studies using rodent models of VCI have made several important findings for understanding the pathophysiology of VCI [49,50,51,52]. Those rodent models replicate representative pathological white matter changes observed in VCI patients, including the disruption of the blood-brain barrier, glial activation, oxidative stress, demyelination with axonal damage, and increase of OPCs in demyelinated lesion (Table 1). Among them, to understand the pathological mechanisms of VCI, a mouse model of prolonged cerebral hypoperfusion achieved by artificially constricting bilateral common carotid arteries is now relatively well-accepted to examine white matter pathology [53]. Here, we introduce some key findings from recent studies using the mouse hypoperfusion model, focusing on oligodendrocyte protection and restoration.

Although there is no clinically proven drug that would protect the white matter from damage in VCI, several therapeutic targets have been reported from studies using the mouse hypoperfusion model. A radical scavenger edaravone is now used in clinical settings for treating stroke and amyotrophic lateral sclerosis (ALS) [54,55,56,57,58]. In a mouse model of chronic hypoperfusion, treatment with edaravone ameliorated white matter damage (myelin degradation, oligodendrocyte death, and blood-brain barrier damage) and promoted compensative oligodendrogenesis presumably by inhibiting hypoxia-induced free radicals from attacking cellular components [59,60,61]. In addition, a phosphodiesterase-III inhibitor, cilostazol, which is also used in clinic for peripheral vascular diseases, was proven to be effective for oligodendrocyte protection and restoration in the corpus callosum of hypoperfused mice through the upregulation of cAMP-responsive element binding protein (CREB) signaling and Btg2 (regulator of cell differentiation) gene expression [62,63]. Furthermore, by combining the mouse model of hypoperfusion with pharmacological and genetic manipulation approaches, several promising targets for oligodendrocyte protection and restoration in VCI have been demonstrated, including sphingosine kinase [64], angiotensin II type 2 receptor [65], Na^+^-K^+^-Cl^−^ cotransporter 1 [66], and transient receptor potential melastatin 2 (TRPM2) channel [67].

Comorbidities are not negligible factors for VCI. In fact, hypoperfused mice with type 2 diabetes exhibited a lower proliferation of OPCs [68]. In addition, the proliferation and differentiation of OPCs after cerebral hypoperfusion were also dampened by age, presumably due to the defects in CREB signaling [62]. Furthermore, as discussed in the section above, cell-cell trophic coupling is critical for maintaining proper oligodendrocyte function. Using a transgenic mouse line wherein BDNF expression is downregulated specifically in glial fibrillary acidic protein (GFAP)-positive astrocytes, astrocyte-derived BDNF was shown to be supportive for oligodendrogenesis under the conditions of cerebral hypoperfusion [69]. Another study demonstrated that pericyte-derived bone morphogenetic protein 4 (BMP4) may have an important role in modulating astrogenesis and oligodendrogenesis after white matter damage by chronic hypoperfusion [70]. In addition, recent study revealed that secretome of endothelial progenitor cells would boost oligovascular remodeling in the hypoperfused mice by increasing vascular density and by potentiating the proliferation and maturation of OPCs [71]. All these studies support the idea that the concept of NVU would be useful to understand the mechanisms of white matter dysfunction in VCI. However, it should be noted that the pathology in human VCI is very complicated, and any single rodent model could not suffice to understand the mechanisms in oligodendrocyte damage/repair in VCI precisely [72]. From the translational viewpoint, the usage of multiple animal models should be considered for examining the underlying mechanisms in white matter damage and for the pursuit of therapeutic targets for VCI.

### 3.2. Alzheimer’s Disease (AD)

AD is usually considered as a gray matter disease due to the distribution of hallmark pathological changes known as extracellular neuritic plaques formed by amyloid-beta (Aβ) aggregation and neurofibrillary tangles, which consists of phosphorylated tau protein. Nevertheless, recent imaging studies showed that hyperintensities of the white matter is a good predictor of AD incidence [73,74,75], and that early AD patients with micro- and macro-structural abnormalities in the white matter have higher risk of disease progression [76]. These findings imply that white matter degeneration may also be an important pathophysiological feature for AD.

White matter pathology in AD may be at least partly due to the modification in white matter structure/component by aging. Overall hemispheric white matter volume is known to decrease with age, and a maximum of a 45% decrease in the total length of myelinated fibers was found between individuals of 20 and 80 years old [77]. Although myelin production by oligodendrocytes may continue throughout life, aging causes thinner myelin sheaths and shorter internodes. In fact, the same study also confirmed an appearance of thinner axons in the elderly [77]. Thinner myelin sheaths and smaller axons would result in functional deficits due to conduction failure and a greater vulnerability to trauma, oxidative stress, or Aβ toxicity [78]. Demyelinated lesions tend to distribute within the areas with relatively low CBF, which are usually found in profound, periventricular white matter. Both in normal elderly populations and in AD patients, a decrease of vessel density in the periventricular region was observed [79], which would eventually lead to oligodendrocyte damage by hypoxic stress. Besides decreases in CBF, a recent pathological study reported that parietal white matter degeneration in AD patients could partially be attributed to Wallerian-like degeneration, which is a secondary axonal loss due to neuronal loss caused by the accumulation of phosphorylated tau in the cortex [80]. Another recent translational study described the predominance of microglia-induced neuroinflammation in the white matter of aged mice/humans and also in the brains of early-onset AD patients [81], suggesting that the evaluation of white matter inflammation may have a clinical value for predicting the onset and progression of aging and neurodegeneration caused by AD. A recent large cross-sectional study of cognitively unimpaired elderly subjects also revealed that a not robust but statistically significant increase in white matter degeneration (hyperintensity in MRI) was observed in subjects with decreased cortical thickness regardless of cortical Aβ accumulation [82]. Serologically, lower levels of Aβ in cerebrospinal fluid and higher levels of plasma Aβ were shown to be associated with the presence and progression of white matter hyperintensity in non-demented elderly individuals [83,84]. These studies suggest that white matter degeneration may be an earlier manifestation of Aβ deposition rather than the degeneration of gray matter. Taken together, white matter damage may be one of the important characteristics of AD patients. Still, a comprehensive analysis of the affected areas within the postmortem brain tissue from AD patients should be performed for pathological verification.

When white matter changes occur in AD patients, it is natural to think that oligodendrocyte lineage cells are likely to be affected within that lesion. In fact, changes in oligodendrocytes were confirmed in human AD brains and in mouse models. In postmortem human white matter tissue, the population of Olig2-positive cells (immature oligodendrocytes) was decreased [85]. Observation of the para-hippocampal white matter also revealed that the diameter of oligodendrocyte nuclei shortened while the mean diameter of neuron nuclei was not affected [86]. On the other hand, one study reported an increase of microtubule-associated protein-2 (MAP2)-positive myelinating oligodendrocytes near the periventricular white matter lesions and a higher number of platelet-derived growth factor receptor (PDGFR)-α-positive OPCs within the degenerated white matter [87]. Therefore, responses of OPCs in AD brains may depend on the diseased conditions/states or brain regions. Analyses in AD mice have also revealed significant functional changes in white matter oligodendrocytes. Compared to wild-type (WT) mice, oligodendrocytes in PS1 knock-in mice were more susceptible to glutamate and Aβ. In APP/PS1 mice (6–8 months old), OPCs were found to be increased compared to WT mice [85,88]. These studies suggest that the abnormalities of oligodendrocyte lineage cells could occur early in the disease course with the presence of presenilin-1 (PS1) mutation. In addition, another study reported that triple transgenic mice (3xTg-AD; 6 months old) exhibited lower myelin basic protein (MBP) expression and a smaller number of myelinating oligodendrocytes but a larger number of non-myelinating oligodendrocytes [89], indicating that AD pathology may impair the endogenous restorative responses in oligodendrocyte lineage cells. Even though neuritic plaques are rarely found in the white matter, oligodendrocytes of the white matter are likely to be exposed to soluble Aβ, which is known to increase in the white matter of AD patients [90]. So, how do Aβ and tau affect oligodendrocytes? Previous studies showed that Aβ was toxic to oligodendrocytes [89,91,92,93]. In rat primary oligodendrocyte cultures, Aβ treatment induced oxidative stress, which then caused mitochondrial DNA damage leading to oligodendrocyte death and dysfunction [94]. Tau protein is toxic to neurons as it disturbs microtuble stabilization. Although severe tauopathy occurs mostly in gray matter, calpain2 (an indicator for axonal loss) was found to be increased in the white matter of AD brains in association with the increase of cortical phosphorylated tau and Aβ [80].

Although it is assumable that oligodendrocyte lineage cells are activated in response to oligodendrocyte damage in AD brains, mechanisms of oligodendrocyte regeneration during the course of AD pathology are still understudied. To the best of our knowledge, pharmacological studies focusing on the remyelination of the white matter in AD patients/animal models is not yet available. However, non-pharmacological therapeutic approaches towards AD have offered us hints to consider effective strategies to promote oligodendrocyte regeneration and remyelination in AD brains. For example, recent clinical data have shown that physical activity is inversely associated with the progression of AD [95]. Also, in basic studies, AD model mice treated with forced exercise showed an attenuation of disease-related pathology. These results were partly attributed to the protection of the capillaries to secure enough blood supply for the white matter [96,97,98,99,100]. Since oligodendrocyte-endothelium interaction in the oligovascular niche is important to sustain angiogenesis and oligodendrogenesis [101], non-pharmacological therapies such as exercise could be novel candidate methods to boost the endogenous compensative responses of oligodendrocytes in AD brains. Nonetheless, in-depth investigations into the underlying mechanisms of oligodendrocyte damage and repair within the disease course of AD would be necessary in future experiments.

### 3.3. Multiple Sclerosis (MS)

MS is characterized by an inflammatory reaction of various types of immune cells against myelin sheaths and oligodendrocytes in the CNS. Eighty percent of MS patients initially develop a clinical pattern with periodic relapses followed by remissions [102]. Since different anatomic areas are involved when relapse occurs in most cases, clinical manifestations vary in each instance. Relapses are nearly always followed by remission to some extent, although recovery to baseline status is often incomplete. Corresponding with the recovery of clinical symptoms, the remyelination of demyelinated lesions has been pathologically confirmed in human brains [103,104,105]. However, remyelination is usually restricted to the lesion borders, and remyelinated internodes do not regain their original length and thickness [106,107,108]. It is also reported that remyelination in acute demyelinated lesions is more efficient than that in chronic lesions [104,109]. These sequences are considered to induce chronic pathological loss of myelin and extensive axonal injury [110].

Attributable mechanisms for the incomplete (re)myelination in MS are predicted to be related to the reduction of the recruitment/maturation of OPCs [111,112,113]. In experimental autoimmune encephalomyelitis mice, NSPCs increased within the SVZ and migrated towards demyelinated lesions for differentiation [114]. However, the failure of migration and the differentiation of OPCs often occur under the conditions of MS, due to a number of inhibitory factors in the lesion microenvironment [115,116]. While numerous mechanisms are involved in the demyelination/remyelination of MS, several important mechanisms may be found in the cell-cell interaction between oligodendrocyte lineage cells and neighboring cells. Under the condition of MS, microglia would be repeatedly activated (polarized). Those activated microglia produce excessive levels of toxic factors, including reactive oxygen species and inflammatory cytokines that exacerbate oligodendrocyte damage [117,118]. In addition, the phagocytotic function of microglia would directly cause oligodendrocyte loss. However, the role of microglia in oligodendrocyte function may not be so simple. The removal of myelin debris by microglia is an important step for remyelination. Also, under some conditions, the preservation of myelin homeostasis would be increased by M2 microglia which promotes OPC differentiation [119,120]. In addition, when exposed to tumor growth factor β (TGFβ)/interferon γ (IFNγ), phenotype changing would occur in microglia to promote oligodendrogenesis [121,122,123]. Similarly, reactive astrocytes may exhibit both detrimental and beneficial effects to oligodendrocytes in MS. As mentioned in the above sections, astrocytes have positive roles in OPC proliferation/differentiation under some conditions. However, in an lysophosphatidylcholine (LPC)-injection white matter injury model, astrocyte-derived endothelin-1 (ET-1) was identified to negatively regulate the differentiation and remyelination of OPCs by promoting Jagged1 expression, which activates the Notch signal in OPCs [124]. While the underlying mechanisms remain to be fully elucidated, the dual roles of microglia and astrocytes in oligodendrocyte regeneration in MS may partly depend on the activation status (i.e., polarization conditions) and/or the distance of those cells from the lesion site [125,126].

## 4. Conclusions

**Figure 2:**
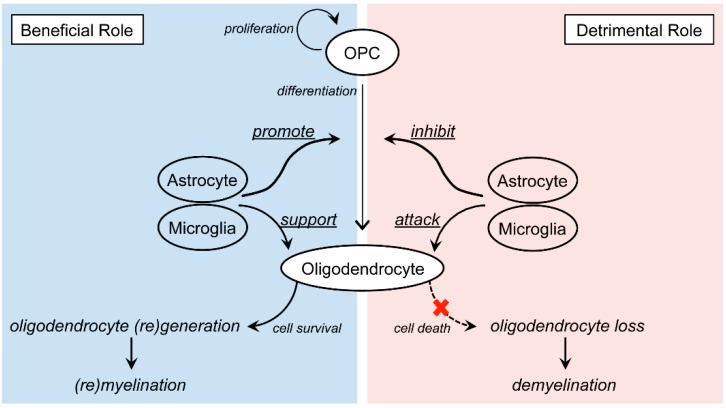
Schematics of the dual roles of astrocytes/microglia in oligodendrocyte damage/repair in central nervous system (CNS) diseases: In response to oligodendrocyte loss, residual oligodendrocyte precursor cells (OPCs) proliferate and differentiate into mature oligodendrocytes. Depending on the surrounding atmosphere, astrocytes and microglia exhibit both beneficial and detrimental roles towards this compensative response. A deeper understanding of their dual roles is important to develop effective therapies towards white matter-related diseases.

The damage of the white matter oligodendrocytes is one of the major characteristics of several neurodegenerative diseases. Under the diseased conditions, multiple deleterious cascades are predicted to be activated to cause the death of oligodendrocytes. Since the simultaneous blocking of all of these cascades is virtually impossible, promoting oligodendrogenesis from OPCs/NSPCs could be one of the effective ways to protect and restore the white matter from CNS diseases. However, guiding OPCs/NSPCs towards efficient remyelination would not be easy. As discussed, successful oligodendrogenesis cannot be achieved by oligodendrocyte lineage cells alone, and the inclusion of many other types of neighboring cells is essential for the process. It should also be noted that under such diseased conditions, those neighboring cells could in turn become deleterious for oligodendrogenesis depending on the surrounding atmosphere (Figure 2). Ultimately, as the concept of NVU originally emphasizes, the comprehension of oligodendrocyte-related cell-cell interaction is important to develop an effective therapy for white matter-related diseases. Further investigations are warranted to examine how oligodendrocyte lineage cells interact with neighboring cells under physiological and pathological conditions.

## Figures and Tables

**Table 1:** Rodent models for vascular cognitive impairment syndrome (VCI).

Parameter	SHR-SP (Rat)	BCAO (Rat)	BCAS (Gerbil)	BCAS (Mouse)
Operation/surgery	No surgery	Ligation only	Coil placement	Coil placement
Cerebral blood flow (CBF) decline (%)		50–70%	~70%	~70%
White matter legion	~20 weeks	~1 week	over 8 weeks	~2 weeks
Cognitive dysfunction		~4 weeks	~2 weeks	~4 weeks

SHR-SP: spontaneously hypertensive rats (stroke prone); BCAO: bilateral common carotid artery occlusion; BCAS: bilateral common carotid artery stenosis.
